# Case of a Gun-Shot Wound in the Back, Followed by Hæmaturia, but No Other Alarming Symptom

**Published:** 1845-10

**Authors:** J. W. Stout

**Affiliations:** Nashville, Tennessee


ART. V.—*Case of a Gun-Shot Wound in the Back, followed by Hœmaturia, but no other alarming symptom.* Read before the Medical Society of Tennessee at the Annual Meeting in May, 1845. By J. W. STOUT, M.D., of Nashville, Tennessee.

Hæmaturia following a wound in the region of the kidneys, the bladder, or the urethra, generally warrants the conclusion that one of these organs has been injured. During the early part of the last year I was called to visit a young man, about 21 years of age, who had received a gun-shot wound; he was very robust and muscular, rather inclining to corpulency. Upon examination I found that the ball had entered on the right side of the spine, below the ribs at the superior and posterior part of the right lumbar region, at a point behind which the kidney of that side is situated; he complained of great pain at the seat of the wound; he was in the erect posture at the time he received the wound. Upon probing it, the direction of the ball was ascertained to be not directly inward, but between the layers of the abdominal muscles. His wound was dressed, and an anodyne administered, which somewhat composed him, but did not induce sleep. The day following I visited him in conjunction with Dr. F. Robertson and Dr. Smith, and found him without arterial excitement, or any alarming symptom whatever. We ordered warm fomentations to the wound; about 10 o'clock he evacuated his bladder for the first time since the receipt of the injury, and instead of urine the fluid discharged was blood, about the same in quantity as an evacuation of urine would have been after such delay. Still no other alarming symptom presented itself; we prescribed emollient drinks, and nauseating doses of tartarised antimony; his urine during the whole of this day was tinged with blood, but on the day following this symptom entirely disappeared, and his bowels were moved without assistance; the wound looked healthy, but did not suppurate until the subsequent day. His recovery, after the suppuration commenced, was very rapid. The lodgment of the ball was never ascertained satisfactorily; but he has not experienced any inconvenience from it since his recovery.

Could the hæmaturia, in this instance, have been occasioned by a wound of the kidney? It cannot be supposed that it resulted from an injury of the bladder or urethra. From the rapidity of his recovery, and the fact that the symptom indicative of wound of the kidney existed for a single day only, as well as the absence of all abnormal indications in the urine after this time, I believe that the kidney was not wounded. This opinion is still further strengthened by the consideration that in a wound of this viscus, the patient can hardly escape extravasation of blood or urine, yet in the case reported not the slightest indication of either of those conditions existed. It is true that blood might have been extravasated in this region without the occurrence of any of those urgent symptoms which follow it in other abdominal regions, since the irritation which it induces in the peritoneum in parts covered by that membrane could not have existed, the kidneys being behind and uncovered by the peritoneum; but extravasation of urine is everywhere followed by the worst effects, and is productive of great danger to the patient.

From the proximity of the kidney to the seat of the wound might not the concussion occasioned in that viscus have been the cause of the discharge of blood? Might it not have occasioned a rupture of some of the small vessels of that highly vascular organ? if not so, might not the congestion induced in it by its nearness to the seat of injury have occasioned a rupture in some of its delicate vessels, the hemorrhage thus induced affording relief to it, and preventing any bad effects that might otherwise have ensued, thus affording an instance of nature interfering to effect what art and sometimes fruitlessly undertakes?

May 1st, 1845.

